# Challenges in Retaining Research Scientists beyond the Doctoral Level in Kenya

**DOI:** 10.1371/journal.pntd.0000345

**Published:** 2009-03-31

**Authors:** Lynette Isabella Ochola, Evelyn Gitau

**Affiliations:** Kenya Medical Research Institute (KEMRI)/Wellcome Trust Collaborative Research Programme, Centre for Geographic Medicine (Coast), Kilifi, Kenya; London School of Hygiene & Tropical Medicine, United Kingdom

## Introduction

An important goal of modern-day African governments should be to develop a sustainable research culture in higher education in order to provide human resources and expertise toward better health and scientific national policies. Regrettably, research in Kenya is mainly funded by Northern collaborators, with the Kenyan government spending only 6.2% of total government expenditure on health in 2001 [1], and even less on health-related research. As a result, the local institutions are not carrying out the bulk of research in the country; instead, most research conducted in Kenya is funded by Northern collaborators: for example, Kenya Medical Research Institute (KEMRI) programs are funded by the Wellcome Trust (United Kingdom), Centers for Disease Control and Prevention (United States of America), and Walter Reed Army Institute of Research (United States of America). These partnerships have contributed to the changing landscape of research in Kenya, and they continue to play an important role in training local scientists. Ongoing programs and projects culminating from these partnerships have significant components designed to build individual and institutional national capacities in a variety of disciplines at all levels. One of the ways this has been done is to provide postgraduate training to young scientists to the doctoral level both at local and overseas academic institutions. However, the issue of capacity retention following training has not been comprehensively tackled. In this Viewpoint, we highlight three competitive doctoral tracks available in Kenya and how the choices students make ultimately play a role in their search for postdoctoral training. Our Viewpoint is related to A. I. Leshner's Editorial in *Science* last year, which focuses on a change in American and British government funding strategies toward new investigators in research [2].

## Capacity Building at the Doctoral Level

Our first example of training in Kenya at the doctoral level is based on the needs of the researcher's home institute. In this instance, the institutions use existing collaborations with Northern partners to secure funds to train students to enable technology transfer. This track is mainly project-driven, and at the end of their training young researchers are expected to return to their home institute. At the end of the training, though, there is a lack of an enabling environment: e.g., the laboratory facilities do not support the introduction of cutting-edge technology, therefore the skills that have been acquired cannot be transferred to the South. Moreover, the pay is not commensurate with their training due to the low demand for their new skills and higher qualifications, and hence they may opt to return to the North for better-paying jobs. For example, as a research assistant, the second author trained in a Northern collaborating institute to use de novo technology, with a Ph.D. as an incentive. Like other scientists found in this position, she opted to change an aspect of her research to advance her postdoctoral training in the local institutes upon her return. Both authors have also found that on returning to local institutions, there is pressure to become an independently funded scientist either immediately after receiving their Ph.D. or after their first postdoctoral position, without adequate exposure and mentorship.

The second available track is where the young researcher together with collaborators at the home institute solicits money from external funding agencies. The doctoral project is normally part of a larger study, and the student does the majority of their training/research at the South-based institute. Although the researcher may retain their employment after the project is over, they may find that at the end of their doctoral training they are encouraged to seek their postdoctoral experience elsewhere. This may be due, but is not limited, to the fact that the home institute may not have enough money to maintain postdoctoral training, and also that mentors may feel that there is an added advantage to being exposed to new environments and ideas outside the home institute. The main difficulty for scientists in our Institute who find themselves in this position is identifying mentors in the North. Also, they are not sure of the support they will get when they identify a Northern or external mentor, and there is an added concern of losing the safety net of being in a local institution.

The third track is where young academics are self-sponsored, either by their own means or with assistance from family and other benefactors, such as in the case of the first author who was sponsored for her Ph.D. by the Gates Malaria Partnership. These categories of people feel no obligation to return to and to work for the local institutes, given that the latter played no part in the financing of their study programs.

In our view, capacity retention upon completion of the doctoral degree is hard to achieve in academic and research institutes in Kenya mainly because of the transitional challenges highlighted in the three examples above. Moreover, human nature being what it is, people are inclined to seek more prosperous employment opportunities once they have acquired the necessary qualifications. Increasingly, Northern institutes have become popular destinations for researchers seeking postdoctoral experiences. This is due, in part, to the relatively better service packages—most notably remuneration—and more research flexibility and innovation existing in these countries. Furthermore, researchers in specialised fields may find it difficult to identify local mentorship. Statistics are scarce on the nature and extent of the problem, but, from anecdotal evidence, it is safe to conclude that a significant proportion of all those sent abroad for further studies do not return at the end of their programs.

## Role of Research Partnerships

The importance of training beyond the Ph.D. level has been underscored by the emphasis on and amount of financial support from Northern funding agencies for scientific capacity building. There is still room for improvement such that the postdoctoral training available contributes to the national research agenda. It is, therefore, important to remember the fundamental difference in the health burdens suffered by the North and South, and thus it is not possible for research and training priorities to be the same. For example, Northern institutes tend to work on a few major tropical diseases, while the South gives priority to preventive medicine and health difficulties related to environment and nutrition [3]. Kenya must emphasize research and prioritize the role of science in development. For poignant reminders of the role we need to play, one only need look at the staggering national statistics of the major causes of mortality as of 2002: malaria (5%), HIV/AIDS (38%), tuberculosis (5%), and diarrheal diseases (7%) [4]. This alone should cause us to make the critical quantum leap in our mindsets, whereby solutions to Kenya's health problems are not only initiated from the North but are also Kenyan-born.

The role of the North has been mainly to bring in the funds, provide jobs, and, hence, drive their own research agenda. Additionally, they offer exposure to training in Northern facilities, to international standards of science and research, sustained training from diploma to B.Sc, M.Sc, and Ph.D., as well as the offer of re-entry grants for postdoctoral scientists. Although this has been recognized as necessary for African scientists to make the transition from student to independent investigator [5], Southern academic institutions need to have a real voice in the design and ownership of North–South partnerships [6], clearly articulating their needs to funders and partners, especially at the postdoctoral level. One way this could be done is by offering international pay packages for postdoctoral scientists in local institutions. Nevertheless, for sustainable development together with funding agencies, Southern academic institutions should target more than one level of training to enhance career progression in one institution [7]. Retention of scientists should be encouraged by ensuring that professional development and increase in remuneration is not solely pegged on the acquisition of higher degrees but also on experience and short courses. Additionally, it is imperative that we gain momentum in creating more South–South partnerships (Box 1) and revel in and build upon the wealth, depth, and breadth of expertise we have acquired from our North–South research experiences.

**Box 1. Capacity-Building Initiatives in Africa**

- Initiative to Strengthen Health Research Capacity in Africa (ISHReCa) http://www.mrc.ac.za/researchdevelopment/ISHReCAbrochure.pdf
- African Institutions Initiative (Wellcome Trust) http://www.wellcome.ac.uk/Funding/Biomedical-science/Grants/Other-initiatives
- African Malaria Network Trust (AMANET) http://www.amanet-trust.org
- The Academy of Sciences for the Developing World—Regional Office for Sub-Saharan Africa (TWAS-ROSSA) http://www.nairobi.twas.org
- Health Science Africa Network E-mail: saninfokilifi.kemriwellcome.org
- Kenyan Young Scientists E-mail: keyoungscigmail.com

## Role of Kenyan Scientists

Local scientists should create research questions from the needs of society so as to feed into national policy, contribute to national health research systems, and address public health challenges. For developing countries, the process of embedding research into their health systems requires competent local scientists and a strongly supportive and enabling environment that will allow research communities to grow and deliver research goods that contribute to the health of the public [8]. One way to do this would be to identify research needs in our community in such a way as to help us develop into individual research scientists. Our training within our North–South institutions is not solely a way of empowering ourselves while isolating ourselves from our community. Instead, we should be leaders ready to bring up the next generation of scientists.

## Role of National Institutions

This is the infrastructure that will enable local research science to be preserved and empowered. National institutions are the enabling environment within which to train up, mentor, and produce the next leaders in science in the country. The leaders in these establishments should debunk inferiority or superiority complexes to allow room for development for all levels of scientists. Therefore, they should be flexible and accommodate the new wave of energized and excited younger scientists wanting to be involved in leading the national science agenda. Crucially, the institutions should demand and exercise the highest level and quality of research by having research-driven curricula and well-defined career structures. This can be realised if the government funds newly trained fellows to carry out research in higher education institutions. It will also encourage the higher education facility to become the initiation point for the maintenance and sustenance of world-class national research. For example, in the United Kingdom the National Health Service funds research in universities, hence driving the national research agenda that feeds into health policy. Additionally, a way to encourage research growth and funding in the universities is through collaborations between universities and research institutes or industry in the form of joint appointments of research fellows.

## Role of the Government

Research capacity remains an unmet challenge in the South [9]. This is especially true for sub-Saharan Africa, where health research in most countries has an allocation of less than 0.5% of national health budgets and health budgets are funded with less than 1% of gross domestic product [8]. Additionally, research and development (R&D) intensity (R&D expenditure relative to the size of the national economy) in Kenya is generally <0.3% [10]. The Kenyan government needs to not only realize but also follow through on their commitment to R&D by increasing their financial input into local scientists' research interests and involving them in achieving national R&D goals. Financing of scientists will enable the development of national research and promote and improve the national health agenda.

## Conclusion

The South bears the greatest burden of the world's infectious diseases, and they need to take a lead role in finding appropriate solutions. Currently, the face of research is changing in Kenya and Africa. Since there is now a well-trained scientific human resource base, we are in a better position than ever before to meet the health challenges of our nation (recommendations are highlighted in Box 2). The training achieved in the North should help enhance the quality and amount of research being carried out in Kenya. Kenya and Africa as a whole should emulate developing countries—such as Brazil, Mexico, Argentina, South Africa, Malaysia, China, India, and Thailand—which are already investing in science, have developed mechanisms to promote excellence in research [11], and have successfully managed to translate knowledge received in the North into thriving self-sustaining national research programs.

**Box 2. Key Points for Building Scientific Capacity**

- Investment in locally available postdoctoral training and mentorship
- Local scientists should play a leading role in North–South partnerships
- Enhancement of South–South partnerships
- Ownership of career development to promote national research agenda
- Increased government budget allocation for research in Kenya
- Strengthening research capability in the universities
